# Life Cycle Assessment of Construction and Demolition Waste Management in Riyadh, Saudi Arabia

**DOI:** 10.3390/ijerph19127382

**Published:** 2022-06-16

**Authors:** Husnain Haider, Sulaiman Yousef AlMarshod, Saleem S. AlSaleem, Ahmed AbdelMonteleb M. Ali, Majed Alinizzi, Mohammad T. Alresheedi, Md. Shafiquzzaman

**Affiliations:** 1 Department of Civil Engineering, College of Engineering, Qassim University, Buraydah 51452, Qassim, Saudi Arabia; 381110157@qu.edu.sa (S.Y.A.); sa.alsaleem@qu.edu.sa (S.S.A.); mfanzy@qu.edu.sa (M.A.); m.alresheedi@qu.edu.sa (M.T.A.); m.uzzaman@qu.edu.sa (M.S.); 2 Department of Architecture, College of Architecture and Planning, Qassim University, Buraydah 52571, Qassim, Saudi Arabia; ahm.ali@qu.edu.sa or; 3 Department of Architectural Engineering, Faculty of Engineering, Assiut University, Assiut 71515, Egypt

**Keywords:** construction and demolition waste, waste management, life cycle assessment, SimaPro, zero waste management, waste recycling

## Abstract

Extensive construction augmenting the infrastructure and real estate projects underpin Saudi Arabia’s Vision 2030 of sustainable cities. A part of this struggle involves the transformation of the existing infrastructure together with new construction, which generates a large amount of construction and demolition waste (CDW). In the absence of a structured life cycle assessment (LCA) framework, the waste management companies are planning future scenarios (phased expansions of material recovery facilities to improve the recycling rate) primarily on economic grounds. This study assesses the environmental impacts of the existing and planned CDW management practices of the Saudi Investment Recycling Company in Riyadh City by dint of LCA. Impact 2002+ performs life cycle impact assessment of the base case (45% recycling), four treatments (61, 76, 88, and 100% recycling), and zero waste scenarios. The study demonstrates the benefits of current CDW (mixed soil, concrete blocks, clay bricks, glazed tiles, and asphalt) recycling in terms of avoided impacts of non-renewable energy, global warming, carcinogens, non-carcinogens, and respiratory inorganics potentially generated by landfilling. For the treatment scenario of 100% recycling, CDW conversion into a wide range of aggregates (0–50 mm) can replace 10–100% virgin aggregates in backfilling, precast concrete manufacturing, encasements and beddings of water mains and sewers, manholes construction, non-load bearing walls, and farm-to-market roads. To achieve long-term economic and environmental sustainability, municipalities need to improve source segregation, handling, and storage practices to enhance the existing (45%) recycling rate to 100% in the next five years and approach the zero-waste scenario by 2030. The findings of the present study motivate the generators for source reduction as well as encourage the recycling companies and concerned organizations in the continuous performance improvement of the CDW management systems across Saudi Arabia on environmental grounds, as an addition to the perceived economic benefits.

## 1. Introduction

Construction and demolition waste (CDW) generation drastically increased with exponential population growth and the allied urbanization trends in the 21st century around the globe. To accommodate the soaring urban population in large cities, replacing old and low-rise buildings with high-rise ones generates substantial CDW [1]. Source reduction, recycling, and reuse are the common CDW management strategies, amidst which recycling has been adopted as the desirable practice worldwide [2]. Only in the United States, 600 million tons of CDW generated in 2018 was over twice the municipal solid waste in the same year; encouragingly, more than 75% of that was reused [3]. A large portion (36%) of the total waste consists of CDW in EU countries, whereas most of the countries achieved the set recovery goal of 75% by 2020 [4]. Conversely, 40% of municipal waste generated from urban areas in China is CDW and the country having 10% recycling rate lags the national target of 13% as of 2020 [5]. The Indian construction industry generates 150 million tons of CDW per year, and only 1.3% is recycled [6]. Kim [1] studied the present waste generation and treatment methods, government policies, and stakeholder efforts toward aggregates recycling in Korea and found a motivational drive in all these aspects of CDW management. CDW in the United Arab Emirates (UAE) accounts for 30% of the total waste, most of which is landfilled [7,8]. Due to inadequate recycling and reuse practices in the Kingdom of Saudi Arabia (KSA), 53 million tons per annum of municipal solid waste causes an environmental loss worth 1.3 billion dollars, whereas 30–40% of urban waste is CDW [9,10].

At the global level, the construction sector contributes to air pollution (23%), drinking water pollution (40%), climate change (50%), and landfill waste (50%) [11]. Most of the CDW components can be recycled or reused, but lack of infrastructure and technology limitations culminate the generated waste in landfills, leading to environmental concerns [12]. Rosado et al. [2] identified the following important factors from reported literature that limit CDW recycling in most cases, low fees for landfill disposal, readily available low-cost aggregates, inadequate quality of the recycled aggregates, and ineffective sorting practices at the source. CDW can impact the environment (e.g., climate change, land utilization, impeded ecology, energy resource consumption, natural resources depletion, aesthetic nuisance, and air, water, and noise pollution), economy (international reputation and tourism losses), and public health and social life (e.g., hazards to health, use of public space, proliferation of pests and impact on safety at work) [13,14]. Inadequately managed CDW can lead to the violation of environmental protection and resource conservation undertaking of the United Nations 2030 agenda for sustainable development [15].

Underpinning the KSA’s Vision 2030 of sustainable cities, extensive construction is augmenting the infrastructure and real estate projects. Advancing these ambitious targets, the transformation of the existing infrastructure along with new construction, particularly in large cities (e.g., Riyadh, Dammam, and Jeddah), generates a large amount of CDW in the country. A study reported the generation of 50–60 tons/1000 m^2^ of waste from the new construction and 700–1200 tons/1000 m^2^ from demolishing aged buildings in KSA [10]. In addition to the primary components (mixed soil, sand, and rock/gavel, concrete blocks, asphalt, clay bricks, and glazed tiles), the CDW in KSA contains some fraction of gypsum and plaster boards, painted timber, reinforced concrete, dirt, and steel (bars, poles, and brackets) [16]; this composition matches with the conventional CDW components stated in other case studies [1,2,17]. Although most of these components are non-hazardous, high generation rates, on-site storage issues, landfilling impacts, and allied logistics complicate the management process of CDW [18].

As per the Country Commercial Guide of Saudi Arabia published by the International Trade Administration (ITA), KSA anticipates redirecting 60% (12% recycling, 35% reuse, and 13% treatment) of CDW from landfills [9]. However, the current CDW recycling rates are much lower than the established targets. For example, the Saudi Investment Recycling Company (SIRC) recycles 45% of collected CDW in Riyadh City [16], whereas most of the generated CDW (0.4 million tons per year) in Qassim is being disposed of in 40 landfills spread over the province [16,19]. Another example is Qassim province where only 6% is being recycled and the rest goes to the landfall [16]. Blaisi [20] identified the following primary challenges, which different concerned sectors (academia, policy makers, generators, and landfill operators) are facing regarding CDW management in KSA. Academia is unable to effectively contribute to research on CDW due to limited data and their weak collaboration with the government organizations. Multiple regulators with the sector-oriented approach, duplication of efforts, and lack of clear strategic planning lead to fragmentation at the policy-making level. At the generators’ end, lack of interest, motivation, awareness, incentives, and promulgation of law resulted in ineffective source separation and handling practices. Landfill operators complain about the lack of infrastructure, treatment technology, absence of fee structure, and investment opportunities for sustainable landfill management.

Realizing the impacts of landfilling on the physical environment, a need for natural resource conservation and energy savings, and the allied carbon emissions are the primary drivers of CDW recycling and reuse. An all-encompassing environmental assessment of the existing (base case) and the improvement scenarios facilitates the decision-making process, by establishing environmental, social, and economic rationale, for the CDW management strategies [21,22]. Life cycle assessment (LCA) is a tool for determining the potential environmental impacts of a product or process by considering environmental interactions (e.g., emissions and energy consumption) throughout the product or system’s entire life cycle [23]. The past studies on LCA evaluated different aspects of building construction, such as high-rise buildings [24], green buildings [25], and the impact of different construction materials [26]. Because concrete and asphalt are the primary components of CDW worldwide, aggregates are the most common recycling product due to their easy recovery from different types of CDW and widespread applications in urban infrastructure construction [1,2]. Hossain et al. [27] conducted a comparative LCA of the recycled aggregates with the case of virgin sources and found significant avoided impacts of 65% greenhouse gases (GHGs) and 58% non-renewable energy.

With arid environment and global warming impacts, KSA is already facing climate change challenges [28]. Although the country’s construction industry is booming, large quantities of CDW threaten the environment, natural resources, and the economy by affecting the tourism promotion plan. Aggregates recycling is one of the most promising CDW management options for developing countries. These aggregates are presently being used for a wide range of applications in KSA, including precast concrete manufacturing, backfilling, encasements and beddings of water mains and sewers, internal walls and manholes, non-load bearing walls, and farm-to-market roads [16]. In the absence of a structured life cycle assessment (LCA) framework, the waste management companies in the KSA are planning future scenarios primarily on an economic basis. Appraising the environmental benefits of CDW recycling can help decision makers sustainably plan improved recycling rates, involve more stakeholders, and secure funds through government and public–private partnerships.

With an aim to enrich the waste management guidelines and practices in KSA, the present study performs a detailed LCA of the CDW management system of Riyadh City. In the absence of an existing CDW management framework in KSA, the baseline data collection was a major challenge that the research team overcame during the course of this study. The specific objectives of the study are to: (i) study the existing CDW management practices in SIRC and collect the baseline data about waste generation, collection, and treatment in Riyadh City; (ii) develop potential waste management scenarios, including the base case scenario (BCS) and several recycling scenarios in line with the future plans of SIRC, (iii) perform detailed LCA to evaluate all the developed scenarios on environmental grounds. The findings of the present study motivate the generators of source reduction and the recycling companies in KSA to enhance their capacities.

## 2. Materials and Methods

### 2.1. Study Area and Data Collection

**Figure 1:**
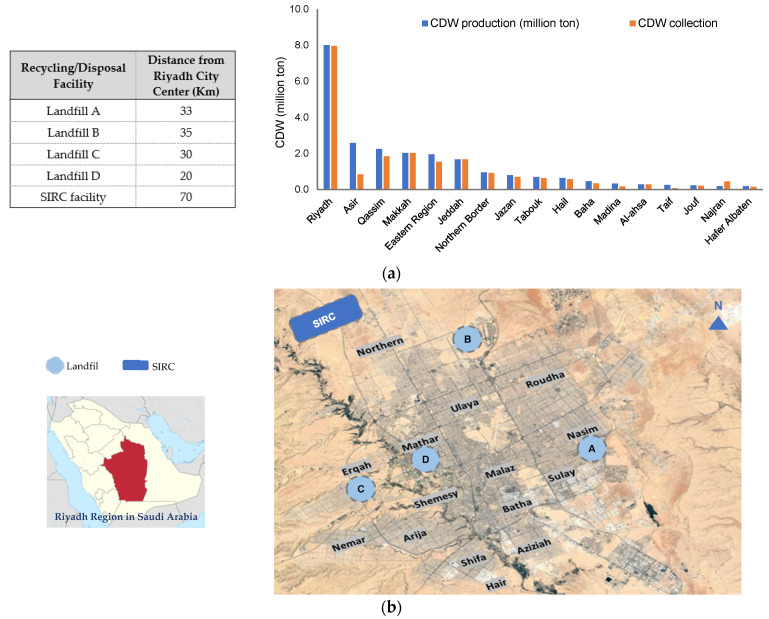
CDW management scenario in Saudi Arabia, (**a**) CDW collection in different regions, (**b**) study area defining the boundaries of Riyadh City and its 15 districts (Letters A–D represents locations of landfills) [16,19].

In order to carry out detailed life cycle impact assessment (LCIA) of a CDW management system, defining the boundaries of the study area is generally the first step. The study area selected in the present research is Riyadh City, which is the capital of Riyadh Region in Saudi Arabia. Riyadh is located at 24.7136° N 46.6735° E with an area of 1973 km^2^ [29]. Presently, the Riyadh Municipality is primarily collecting 100% of the CDW generated from all types of urban land uses, including residential, commercial, public, and industrial. Figure 1a shows that Riyadh City collects the largest amount of CDW in the country, and Figure 1b describes the boundary of the study area and the 15 municipalities covering the city’s districts [16,19].

Presently, the municipalities in Saudi Arabia are facing several challenges related to the current practices of CDW management from the absence of suitable regulation for commitment control of the subcontractors who are responsible for collecting the generated CDW from source to landfill [20]. SIRC is the main recycling facility in Saudi Arabia that is located 70 km north-west of the city center. Figure 1 shows the location of the SIRC facility and the four engineered landfills, which are accommodating the CDW along with the municipal waste. The SIRC [16] and Ministry of Municipalities in Riyadh (MOMRA) [19] were our primary sources of contact for CDW data acquisition for LCA. Presently, the SIRC is collecting 100% of CDW generated in the Riyadh City and produces different types of aggregates from the collected CDW based on their sizes (0–5 mm, 5–10 mm, 10–20 mm, 20–50 mm). These aggregates will be used for backfilling, pipes backfilling, construction of precast concrete, construction of concrete manholes, manufacturing of hollow and solid bricks, concrete for infrastructure installations, sub-base layers, temporary farm-to-market roads, and pipe bedding and surroundings.

### 2.2. Life Cycle Assessment

LCA is a 21st century cutting-edge tool for evaluating the environmental impacts of any development activity throughout its life cycle, initiated from selection and production of raw materials, to process design, construction, and operations, to end of life. Nevertheless, recycling and reuse of materials are aspects that need to be considered in order to have an eco-friendly building environment. During the design phase, selection of materials is directly related to the waste generated for the same building during the construction and demolition phases. The LCA concept assesses the effect of environmental conditions during the completion of the life cycle of a building [30]. The present study adopted the following steps, given in the ISO 14040 [31] and ISO14044 [32] standards, and developed by the International Organization of Standardization (ISO): (i) define goal and scope definition, (ii) prepare life cycle inventory (LCI) preparation, (iii) perform life cycle impact assessment (LCIA), and (iv) interpret the LCIA results.

#### 2.2.1. Goal and Scope Definition

General public agencies working for waste management and the municipalities concerned with stormwater deterioration from dumping sites directly benefit from an efficient CDW management system. In the absence of a structured LCIA process in the country, the study’s findings will be highly useful for the researchers, consulting engineers, and operations managers. The present study used the latest version of SimaPro^®^ 9.3.0.3 (faculty license) software for LCA. The software is based on an attributional approach that describes all the potential environmental impacts over the life cycle of a system [33]. The background methodology of SimaPro contains rational and measurable data of past studies along with all the waste management processes (e.g., storage, handling, sorting, and disposal) (EC 2010). The primary data used to develop the life cycle inventory were obtained from MOMRA website, through personal visits to SIRC, and the published reports. Impact 2002 + v.2.15 evaluated the life cycle impacts of CDW using midpoint and endpoint categories.

#### 2.2.2. Life Cycle Inventory

Past studies reported no significant difference between the results of Impact 2002 versions for region specific analysis and Centrum voor Milieukunde Leiden (CML) baseline for global average impact [2]. Therefore, the Impact 2002 + v2.15 library for a European context is used because normalization factors for Saudi Arabia are not available. In the geographical context of the study area, the European method was more rational than the global and North American methods available in SimaPro software. This assumption is consistent with the past practice of using Impact 2002 for the study areas not having specific factors provided in SimaPro, for instance a recent study conducted by Rosado [2] on LCA of CDW management in Brazil. Ecoinvent v.3.1 database obtained the inventory database, which included direct and indirect burdens in terms of material and energy needed for CDW management processes. Some irrelevant burdens were avoided for the specific scenario of the study area. For instance, as SIRC has already reached 100% waste collection in Riyadh City, transportation impacts were excluded from scenario analysis.

According to the MOMRA, the total production of CDW is 5.3 million tons per year, as of 2020 [19]. Table 1 presents the composition and quantities of the CDW in Riyadh City. The CDW management infrastructure at the first recycling facility operated by SIRC can efficiently handle and process 3.6 million tons per year, which is around 70% of the estimated CDW by the MOMRA. However, SIRC found a higher production of around 8 million tons per annum (MTPA) based on their investigations and assumptions. This way SIRC infrastructure is presently taking care of 45% of 8 MTPA. The company also has estimated around a 3% annual increase, which will lead to a total CDW production of around 10 million tons per year by 2025. Consequently, they are making attempts to increase the size of their infrastructure to accommodate the projected waste. SIRC receives the waste (3.6 MTPA) collected by all the 15 municipalities shown in Figure 1 and effectively recycles 90% (3.24 MTPA), while the remaining fraction safety is disposed of in the landfill.

**Figure 2:**
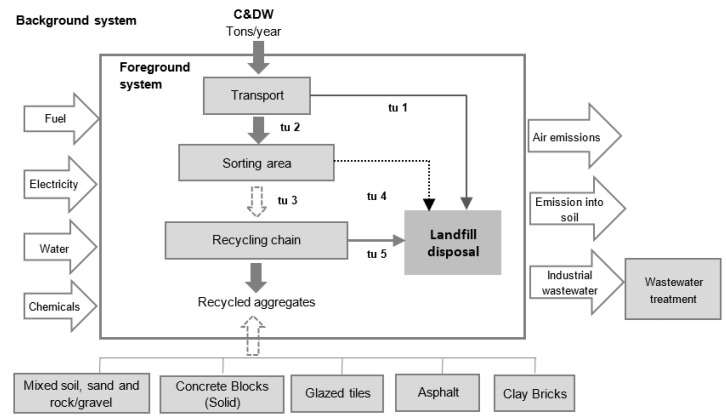
System boundaries for the municipal CDW management systems considered in this study, with the indication of the foreground and background systems. Dashed lines refer to the streams that have differences among the management systems analyzed. The transport stages are indicated by the acronym “tu” (transport unit).

Figure 2 presents the existing process at the SIRC facility, which consists of transport, sorting, recycling, and production of aggregates. The inputs to the processes are fuel, electricity, water, and chemicals in addition to CDW. Presently, 45% (3.6 MTPA) of the generated waste (8 MTPA) consists of (i) mixed soil, sand, and rock gravels, (ii) concrete blocks, (iii) glazed tiles, (iv) asphalt, and (v) clay bricks. The remainder (4.4 MTPA) of the generated waste goes directly to the landfill. After sorting, 90% of these materials are converted into recyclable aggregates and the remaining 10% goes to the landfill. The outputs of the foreground processes are air and soil emissions. The wastewater primarily contains sediments which are being removed through the gravity settling process. The system expansion method was used to avoid the allocations problem in LCA model. LCI of the processes or products replaced by the obtained co-products was subtracted from the analyzed system [33,34].

#### 2.2.3. Life Cycle Impact Assessment

LCIA recognizes and evaluates the extent and importance of the studied system’s potential environmental impacts. This phase involves the accounting and evaluation of potential environmental impacts generated by the product by categorizing and characterizing the flows. Impact 2002 + v.2.15, as described in [35], has been used to evaluate the environmental impacts related to LCA. Past studies also suggested the use of Impact 2002 + v.2.15 in the absence of local data [2,3,36,37,38,39]. Due to the lack of CDW data for LCIA in Saudi Arabia, different waste management scenarios (described in the following section) were compared and analyzed using midpoint and endpoints categories. Single score midpoint categories were carcinogens, non-carcinogens, respiratory inorganics, respiratory organics, terrestrial ecotoxicity, terrestrial acid/nutria, land occupation, global warming, and non-renewable energy, whereas the single score endpoint categories included natural resources, climate change, ecosystem quality, and human health.

Most of the direct burdens for the life cycle inventory were obtained from official reports or technical visits to infrastructure. The remaining direct burdens, indirect burdens related to materials and energy required for CDW management operations, and avoided burdens have been obtained from the literature and Ecoinvent v.3.1 database that was updated with site specific data where possible. The allocation problem in the LCA model was avoided by using the system expansion method (also called ‘‘avoided burden” or ‘‘substitution”), in which the life cycle inventory of the processes or products replaced by the obtained co-products is subtracted from the analyzed system [33,34].

#### 2.2.4. Interpretation

Interpretation of LCIA results is the last stage of the LCA. The LCIA results were compared for all the scenarios to come up with the best-case scenario. The scenarios developed in the present study are described in the following section.

### 2.3. Development of CDW Management Scenarios

**Figure 3:**
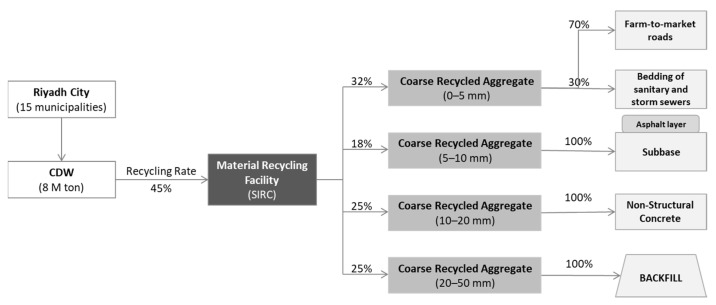
Overview of recycling process in Riyadh BCS.

**Figure 4:**
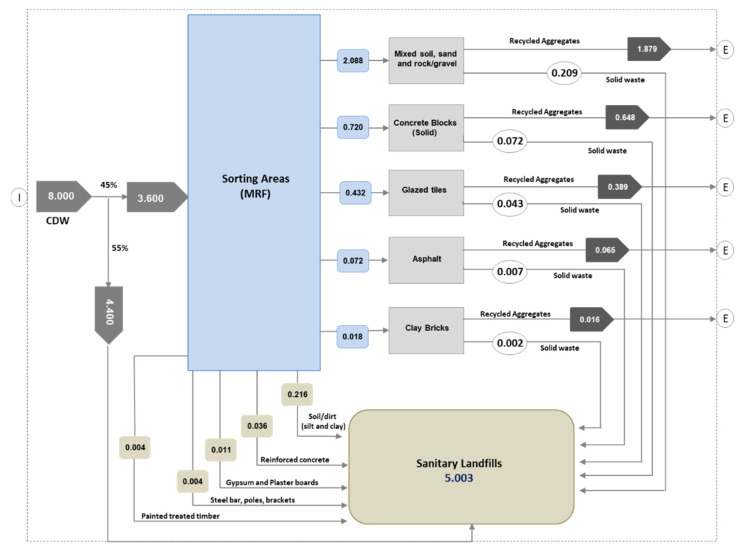
Material flow analysis of CDW management system for the BCS indicating main inputs (I) and exits (E). Data are expressed in million tonnes.

Figure 3 illustrates the BCS of CDW handling and management practices in Riyadh, where the overall recycling rate is 45%, from which 10% is rejected from the material recovery facility (MRF) and joins 55% non-recycled waste in the landfill. Figure 4 describes the material flow analysis for the CDW management system practiced in the BCS. To achieve maximum outcomes of the present research, we aimed at LCA of the scenarios planned by SIRC that have higher probability of being practically implemented in comparison to hypothetical scenarios. As of 2021, Riyadh City generated 8 MTPA and expected to receive 10 MTPA by 2025 with the 3–4% increment per year. In order to handle the projected waste, SIRC plans to enhance the capacity of their facility to 10 MTPA by 2025 with 100% recycling.

In view of the ambitious future plans of SIRC, five treatment scenarios (TSs) were analyzed to improve the existing CDW management practices in Saudi Arabia. Table 2 describes the six scenarios, including five treatments and the BCS, developed in the present research. TS1 to TS4 in Table 2 assess the life cycle impacts of improved recycling rates, according to the company’s plan. In TS1, 61% of the projected waste (8.5 MTPA) by the year 2022 will be recycled to aggregates. With 10% of the recycled waste being rejected, 59.5% of the total waste will be finally dumped into the landfill. Likewise, the next three scenarios evaluate the projected plans for 2023 to 2025 with further increase in recycling to 76% in the case of TS2, 88% in TS3, and 100% in TS4. All these scenarios will reject 10% of the recycled waste that will go to the landfill along with the non-recycled CDW. Finally, zero waste scenario (ZWS) converts 90% of the waste to aggregates and uses the remaining 10% for energy production without producing any reject to the landfill. Hence, the recycling rate of ZWS is 100%, achieving climate neutrality. SIRC was found in 2017 and practically started CDW management in the beginning of 2020 [16]. Although the overall master plan of SIRC seems to be overambitious, the past performance of the company to achieve 100% collection with 45% recycling in the last few years motivated us to evaluate the planned scenarios based on the LCA. The LCA results will help in decision making, encompassing both the economic and environmental dimensions even in case of unexpected delays in achieving the planned scenarios.

## 3. Results

### 3.1. LCIA of Base Case Scenario

**Figure 5:**
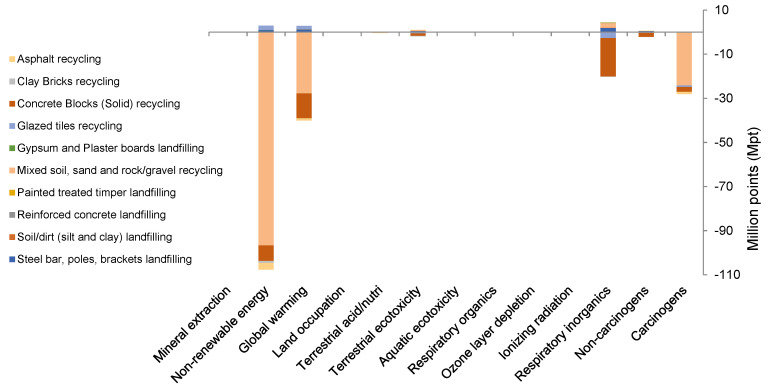
Normalized results of impact assessment of the BCS of construction and demolition waste management system in Riyadh City obtained by using normalized factors for Europe of Impact 2002+ methodology. The vertical axis shows the percentage increase or decrease in life cycle impact categories due to recycling of the particular type of CDW, in comparison to the case of its direct disposal.

In the midpoint categories, Table 3 presents that non-renewable energy accounted for 53% in BCS, whereas global warming accounted for 21%, carcinogens accounted for 13%, non-carcinogens accounted for 1.1%, and respiratory inorganics accounted for 10% (with a total of around 98%) of the total impacts in BCS. The cumulative contribution of the remaining categories given in Table 3 is only 2%. Although selecting the European context seems rational due to the neighboring geographical location of Saudi Arabia, the results presented in Figure 5 might differ from the actual results for the study area. Table 3 also presents percentage contributions of endpoint categories. It can be seen in the table that natural resources have the highest contribution (53.2%), followed by human health (24.9%) and climate change (21.2%) impacts, whereas ecosystem quality has the least contribution to environmental impacts amongst the midpoint categories.

Figure 5 illustrates the overall normalized results of LCIA of the BCS of CDW management in Riyadh. The figure indicates the avoided impacts of the existing recycling process in BCS and the negative sign indicates the impact reduction in comparison to the case of direct disposal of the waste. The figure clearly shows that non-renewable energy global warming is the significant midpoint category affected by the CDW recycling, whereas respiratory organics and carcinogens are the primary endpoint categories that received the benefits of recycling.

### 3.2. Life Cycle Impact Assessment of Improvement Scenarios

**Figure 6:**
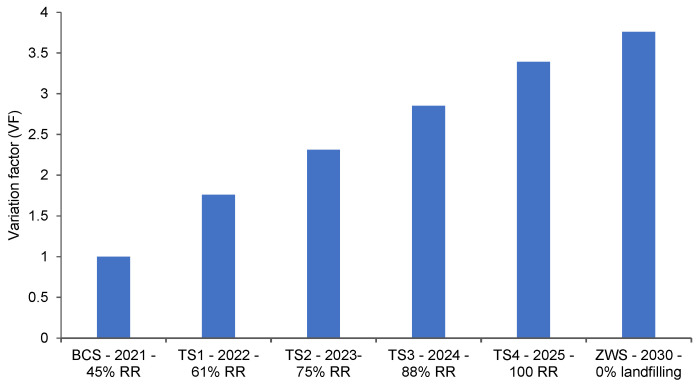
Variation factor for all scenarios with respect to the BCS with variation factor equal to 1. The terms BCS, TS, and RR represent BCS, treatment scenario, and recycling rate, respectively.

**Figure 7:**
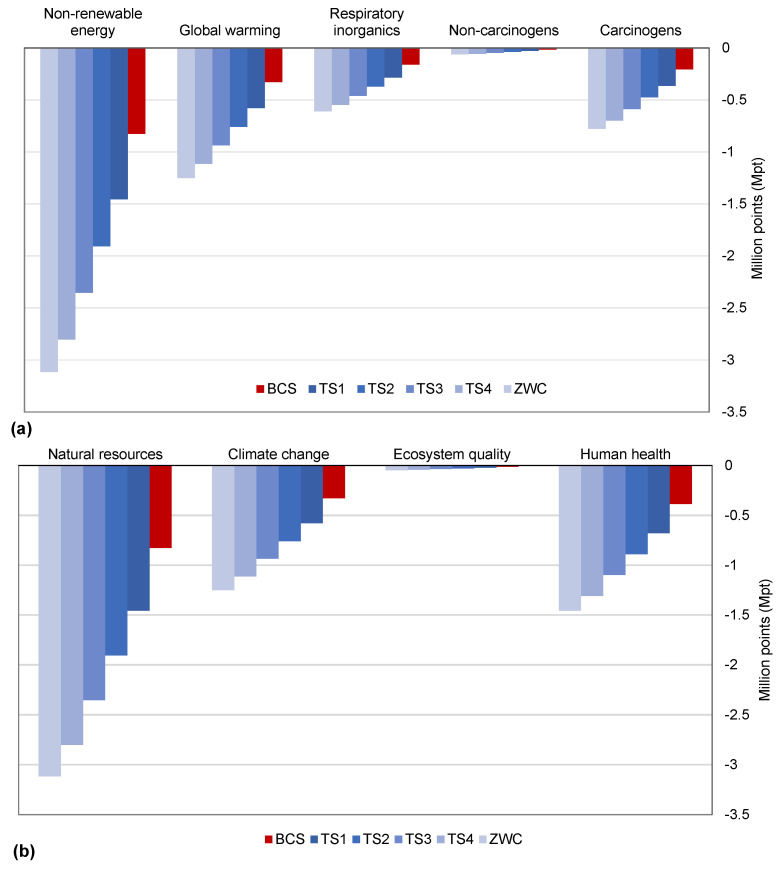
Normalized results of impact assessment obtained by using normalized factors for Europe of Impact 2002+ methodology. (**a**) normalized midpoint results comparing the BCS (BCS with 45% recycling rate in 2021) with TS1 Treatment Scenario 1 (TS1 with 61% recycling rate in 2022), TS2 with recycling rate of 75% by 2023, TS3 with recycling rate of 88% by 2024, TS4 with recycling rate of 100% by 2025 and 10% recycling process waste to landfill, and zero waste scenario (ZWS with 100% recycling and 0% waste to landfill); (**b**) normalized endpoint results for the same treatment scenarios.

To improve the BCS of CDW management in Riyadh City, the present study developed four treatment scenario (to increase percentage recycling) and a zero-waste scenario (see Table 2). For evaluation of different scenarios, sensitivity analysis compared the LCIA results of scenarios with the BCS. The comparison results are reported in term of a variation factor (VF) [40], which essentially is the ratio between the scenario’s results and the BCS. The VF equal to “1” shows no variation, VF less than 1 indicates scenario’s performance worse than the BCS, and the value of VF greater than one reflects improvement by implanting the scenario. The negative value of VF indicates a modification of the potential impact from positive to negative or vice versa. Figure 6 shows the estimated variation factors for all the five improvement scenarios, considering the main impact categories. It can be seen in the figure that VF exceeds a value of 2 after the increasing recycling rate (RR) to 75% in treatment scenario 2 (TS2) from the existing recycling rate of 45% in the BCS. A further increase in recycling, increasing VF to around 4 times higher than the BCS, manifests a multifold reduction in environmental impacts through CDW recycling. Figure 7 presents the normalized results of impact assessment on all the CDW waste management scenarios described in Table 2, obtained from normalized factors for Europe of the Impact 2002+ methodology.

## 4. Discussion

As of 2021, the material recovery facility received 5.3 MTPA constructions and demolition waste collected from Riyadh City, which is 66% of total (8 MPTA) generated waste. The facility is expecting to receive 10 MTPA (100% of the generated waste) by 2025. Presently, 45% of 5.3 MPTA (2.4 MTPA) is being recycled, whereas the remaining 55% is directly being dumped into the landfill. The recycled material, including mixed soil, sand, and rock/gravel (MSSR), CBS, glazed tiles, asphalt, and clay bricks, is 92.5% of the collected CDW. Recycled aggregates recovered from this significant portion of CDW can play an essential role in replacing primary aggregate needs for infrastructure development, e.g., roads and concrete [1,20,41]. The past studies achieved the same properties of concrete with recycled aggregates as of natural aggregates with allied environmental benefits [42,43]. The remaining non-recycled waste consisting of dirt (silt and clay), reinforced concert, gypsum and plaster boards, steel (bars, poles, and brackets), and painted timber is a small portion of total generated CDW. Based on the existing MRF capacity, the recycled material is segregated from the total collected 5.3 MPTA. The segregated recycled material then passes through the picking station with magnets separating the remaining portion of steel and the density separator removes small-sized and fine particles which are not suitable for aggregates formation. This waste counts for around 10% of the recycled waste and also joins non-recycled waste in the landfill. The first four treatment scenarios (TS1–TS4) essentially improve the existing recycling rate from 45% to 100% with the same process chain, in which 10% fines will go to the landfill by 2025. The MRF is committed to achieving the zero waste scenario, in line with the KSA Vision 2030, by using the fines in concrete manufacturing in the future.

**Figure 8:**
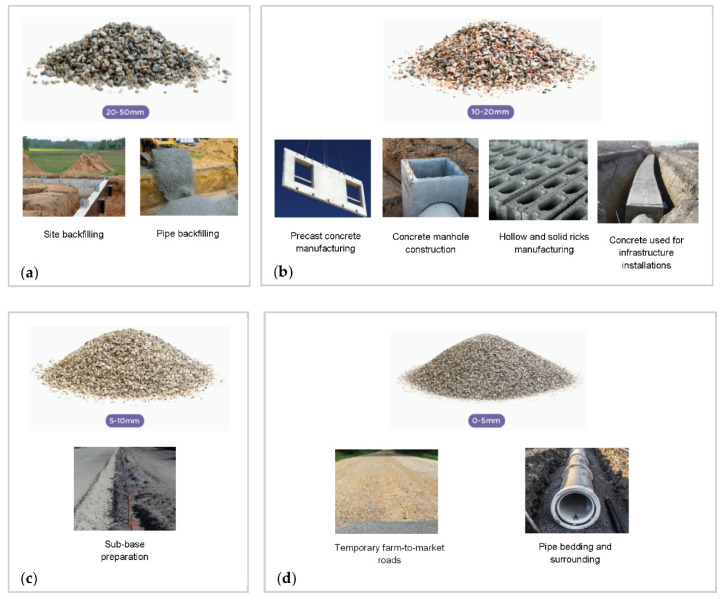
Pictorial vignettes of recycles aggregates and their potential uses, (**a**) 20–50 mm aggregates, (**b**) 10–20 mm aggregates, (**c**) 5–10 mm aggregates, and (**d**) 0–5 mm aggregates. (Source: SIRC [16]).

Figure 8 presents the pictorial vignette of the four types of aggregates (classified based on their sizes) produced through CDW recycling at the Riyadh’s MRF and their potential uses. Recycled aggregates generated from 100% recycling (TS4) can replace 10% to 100% virgin aggregates in several manufacturing and construction activities. Figure 8a shows that the largest size of aggregates produced is 20–50 mm, which can replace up to 100% the virgin aggregates required for all backfilling applications, such as raising the site level, retaining site structure, and filling excavated areas. The same aggregates can also replace (100%) of the virgin aggregates needed for covering pipe networks in the study area (see Figure 8a). The next size of aggregates, 10–20 mm, can replace up to (i) 10% of virgin aggregates in the manufacturing of internal walls, (ii) 10% of virgin aggregates in concrete for the manufacturing of manholes of sanitary and stormwater drainage systems, (iii) 25% of virgin aggregates for the manufacturing of hollow bricks to be used in non-load bearing walls, and (iv) 100% of virgin aggregates for the encasement of water and sewerage linear assets.

The next smaller size, 5–10 mm, of recycled aggregates can replace up to 100% of virgin aggregates in the sub-base of road construction. Finally, the smallest-sized (0–5 mm) aggregates can replace 100% of the virgin aggregates in the construction of temporary farm-to-market roads and as a bedding of water mains and sanitary and storm sewers. Similar practices have been reported for Korea, where 80% of recycled aggregates are used in backfilling (Figure 8a) and road construction (Figure 8c,d) [1]. For concrete applications, the inferior mechanical properties of recycled aggregates, in comparison to natural aggregates, the addition of steel fibers and additives (e.g., fly ash and micro silica) into the concrete mix can enhance the structural properties of concrete [8,44,45].

The MRF at Riyadh City recycles MSSR for construction purposes. This practice significantly reduces the impact on natural resources by minimizing excavation of sand, clay, and rocks. Figure 5 manifests the overall positive environmental impacts of MSSR recycling more than any other process due to its largest fraction in the CDW generated from Riyadh City. MSSR reduced 86% carcinogens in comparison to direct disposal, mainly due to the avoided emissions of C_2_H_3_Cl eq, resulting from the recycling process. MSSR recycling improved 72% global warming potential due to decreasing GHG emissions (as CO_2_-eq) from the recycling processes, instead of landfilling. Furthermore, the 91% avoided impact for non-renewable energy can be seen in Figure 5 by improving the natural resources’ conservation, and 7% for non-carcinogens, mainly due to the avoided emissions of C_2_H_3_Cl eq resulting from the MSSR recycling process. However, the 11% increase in respiratory inorganics category was observed due to the increase in PM_2.5_ emissions associated to MSSR recycling.

The second most important activity, in terms of life cycle impacts, is the concrete blocks (solid) (CBS) recycling (see Figure 5). CBS recycling positively contributes to carcinogen (8% reduction) and non-carcinogen (80% reduction) categories due to avoided C_2_H_3_Cl eq emissions. This recycling activity at Riyadh MRF also improved 29% global warming and 7% non-renewable energy categories. Furthermore, CBS recycling improved the respiratory inorganics category 96% due to avoided emissions of PM_2.5_ in comparison to the case of direct disposal.

In the BCS, glazed tiles recycling is negatively contributing to global warming (4%) and non-renewable energy (2%) due to increasing CO_2_-eq emissions and natural resources consumption in the recycling processes. The process mitigates the impact of respiratory inorganics by 15% due avoided emissions of PM_2.5_, and carcinogens and non-carcinogens by 2% and 11%, respectively, due to avoided C_2_H_3_Cl eq emissions. Clay bricks recycling also positively contributes (around 1%) to the respiratory inorganics and non-carcinogens categories by avoiding PM_2.5_ and C_2_H_3_Cl eq emissions. Likewise, asphalt recycling contributed positively to carcinogens (3%), global warming (2.5%), and non-renewable energy (3%) categories by minimizing PM_2.5_ and C_2_H_3_Cl eq emissions and conserving natural resources. However, the respiratory inorganics category has been negatively impacted by 1% due to the generation of PM_2.5_ from the asphalt recycling process. The other impact categories can be neglected because of their minute variations as a result of recycling instead of direct landfilling.

Presently, gypsum and plaster boards, painted treated timber, reinforced concrete, dirt, and steel (bars, poles, and brackets) are being landfilled after segregation from the collected CDW. Landfilling of these recyclables negatively contributes (primarily) to respiratory inorganics, global warming, and non-renewable energy categories. Understandably, dirt (silt and clay) dumping releases PM_2.5_ that negatively contributes to respiratory inorganics by 52%, global warming by 16% due to the release of CO_2_-eq emissions, and non-renewable energy by 22% due to the consumption of crude oil (fuel) in the disposal process of dirt to the engineered landfill. Mixed with plasterboards and reinforced concrete, painted treated timber (a small fraction of the total CDW in Table 1) gives minor avoided impacts and commonly goes to landfills. For this reason, source segregation can help timber recycling to achieve higher economic and environmental benefits [2].

Dumping of reinforced concrete increases respiratory inorganics by 36%, global warming by 32%, and non-renewable energy by 20%. Similarly, gypsum and plaster boards dumping contributes to respiratory inorganics by 73%, global warming by 8.5%, and non-renewable energy by 10%, whereas dumping of painted treated timber increases 40% of respiratory inorganics and both the global warming and non-renewable energy by 25% each. Although iron and steel represent a small portion of total CDW (Figure 5), disposal of steel bars, poles, and brackets into the landfill contributes to respiratory inorganics by 37%, global warming by 23%, and non-renewable energy by 16%. Considering the end-of-life phase of buildings, the past studies reported the high significance of steel recycling for the avoided impacts of CDW management [37]. In other words, these impacts can be mitigated through the recycling of these materials instead of the existing landfilling practice.

The LCIA results for all the scenarios illustrate the highest reduction in the non-renewable energy category with an increase in recycling rate (see Figure 7a). Increasing the recycling rate of 45% for BCS to 61% by 2022 will potentially reduce 43% of the impact of non-renewable energy consumption, which will further reach 70% impact reduction in this category by recycling 100% of the generated CDW in Riyadh City. A further 3% impact reduction on non-renewable energy consumption can be observed by recycling 10% waste generated from the recycling process itself. The other important midpoint categories that improved in a similar fashion with increasing recycling rate are global warming, carcinogens, and respiratory organics. A slight improvement in non-carcinogens can also be seen in Figure 7a with an increase in the recycling of CDW.

The LCIA results for endpoint categories shown in Figure 7b also depict the similar impact reductions on natural resources, human health, and climate change with increasing recycling rates by 2030. Carcinogens and respiratory organics are the primary contributors to the human health endpoint category (see Table 3 and Figure 7b). An overall reduction of 70% through 100% recycling is a noteworthy contribution to human health, which can improve Disability Adjusted Life Years (DALYs) associated with CDW in the study area. The lowest contribution of ecosystem quality amongst the endpoint categories in Figure 7b is realistic considering the lack of freshwater ecosystem and the potentially disappearing fraction of species in the study area. As the study adopted the Impact 2002 + v2.15 library for the European context, the inherent uncertainties in results can be evaluated using region-specific normalization factors constrained to their availability.

Based on the findings of this study, a detailed CDW framework will be developed for the long-term sustainability of natural resources in KSA. The proposed framework will provide guidelines to the municipalities and construction companies to follow best practices for resource conservation and environmental protection in line with the 2030 Vision of Saudi Arabia.

## 5. Conclusions

Large cities in the Gulf region are producing a large quantity of CDW annually. Being one of the largest cities and the capital of Saudi Arabia, Riyadh is currently producing over 8 MTPA from 15 municipalities covering all the important regions of the city. To meet the sustainable development goals of KSA Vision 2030, the entire city is going through extensive construction and demolition activities. The present study assessed the potential environmental impacts of the current (and planned) CDW management system of Riyadh City using Impact 2002+ LCIA methodology. The assessment results revealed that MSSR recycling significantly avoided the potential impacts of “non-renewable energy”, “carcinogens”, and “global warming” midpoint environmental impact categories generated by landfilling. The study also found that concrete block recycling significantly avoided the human health impacts of “non-carcinogens” and “respiratory inorganics” generated by landfilling. In addition to the direct avoided impacts of landfilling, conversion of CDW into different sized (0–50 mm) aggregates can replace 10–100% virgin aggregates in several construction activities, including backfilling applications, encasement and bedding of water mains and sanitary and storm sewers, manufacturing of internal walls and manholes, non-load bearing walls, encasement of water and sewerage linear assets, and construction of farm-to-market roads. These results encourage the 15 municipalities in Riyadh City to actively participate in source segregation, handling, and storage of CDW to enhance the effectiveness of MRF operated by SIRC in Riyadh City. With the support of the municipalities, SIRC can effectively improve the existing 45% recycling rate to 100% in next five years and finally progress toward a zero waste scenario in line with the KSA Vision 2030. Undoubtedly, SIRC needs to make every effort to implement its CDW management plans in the given timeframe. In order to attain the environmental benefits of CDW reuse and recycling, the outcomes of the present study need to be validated at each development stage (capacity enhancement, process change, and practices) of the existing facility.

## Figures and Tables

**Table 1:** Composition and quantities of CDW in Riyadh (Source: [19]).

CDW Composition ^a^	Fraction	Amount (Tons per Year)
Mixed soil, sand, and rock/gravel	0.58	4,640,000
Concrete Blocks (Solid)	0.2	1,600,000
Glazed tiles	0.12	960,000
Soil/dirt (silt and clay)	0.06	480,000
Asphalt	0.02	160,000
Reinforced concrete	0.01	80,000
Gypsum and Plaster boards	0.003	24,000
Clay Bricks	0.005	40,000
Steel bar, poles, brackets	0.001	8000
Painted treated timber	0.001	8000
Total	1	8,000,000

^a^ Other types of waste are negligible.

**Table 2:** Construction and demolition waste management scenarios for Riyadh City.

Scenario	Planning Year	Recycling Rate (%)	CDW Disposed to Landfill (%)		
			Direct Waste to Landfill	Reject from MRF to Landfill	Overall Waste to Landfill
BCS: Base case scenario	Present	45	55	4.5 (10% of RW ^1^)	59.5
TS1: Treatment scenario 1	2022	61	39	6.1 (10% of RW)	45.1
TS2: Treatment scenario 2	2023	76	24	7.6 (10% of RW)	31.6
TS3: Treatment scenario 3	2024	88	12	8.8 (10% of RW)	20.8
TS4: Treatment scenario 4	2025	100	0	10 (10% of RW)	10
ZWS: Zero waste scenario	2030	100	0	0 (Climate neutrality)	0

^1^ Recycled waste (RW).

**Table 3:** Percentage contribution of environmental impact categories in SimaPro for BCS.

Midpoint Category	Endpoint Category	Unit	Environmental Impact Contribution %	
			Midpoint	Endpoint
Carcinogens	Human health	kg C_2_H_3_Cl eq	13.3	24.9
Non-carcinogens	Human health	kg C_2_H_3_Cl eq	1.1	
Respiratory inorganics	Human health	kg PM_2.5_ eq	10.4	
Respiratory organics	Human health	kg C_2_H_4_ eq	0.1	
Terrestrial ecotoxicity	Ecosystem quality	kg TEG soil	0.5	0.8
Terrestrial acid/nutria	Ecosystem quality	kg SO_2_ eq	0.2	
Land occupation	Ecosystem quality	m2org.arable	0.1	
Global warming	Climate change	kg CO_2_ eq	21.2	21.2
Non-renewable energy	Natural resources	MJ primary	53.2	53.2
	Total		100	

## Data Availability

All the shareable data are included in the main manuscript.

## Abbreviations

BCS	Base case scenario
CDW	Construction and demolition waste
CML	Centrum voor Milieukunde Leiden
DALYs	Disability Adjusted Life Years
KSA	Kingdom of Saudi Arabia
LCA	Life cycle assessment
LCIA	Life cycle impact assessment
MRF	Material recovery facility
MSSR	Mixed soil, sand, and rock/gravel
MTPA	Million tons per annum
SIRC	Saudi Investment Recycling Company
TS	Treatment Scenario
ZWS	Zero waste Scenario
